# Homocysteine in retinal artery occlusive disease: A meta-analysis of cohort studies

**DOI:** 10.1038/s41598-017-16065-2

**Published:** 2017-11-16

**Authors:** Xuetao Huang, Yezhen Yang, Yiqin Duan, Yi-Qun Kuang, Ding Lin

**Affiliations:** 1 Department of Ophthalmology, Changsha Aier Hospital, Aier School of Ophthalmology, Central South University, Changsha, 410007 China; 2 Department of Ophthalmology, Huaihe Hospital of Henan University, Kaifeng, 475000 China; 3 Institute of Infection and Immunity, Henan University & Centre for Translational Medicine, Huaihe Clinical College, Huaihe Hospital of Henan University, Kaifeng, 475000 China

## Abstract

Few studies have reported the relationship between retinal artery occlusion (RAO) and plasma homocysteine (Hcy) levels. Our goal was to evaluate the association between the plasma Hcy level and the risk of RAO disease. Several databases were searched for all published studies that involved Hcy and RAO. Six studies evaluated hyperhomocysteinemia (hHcy) in retinal artery occlusion patients and controls; the incidence of hHcy in patients with RAO was higher than the control and the pooled odds ratio (OR) was 6.64 (95% confidence interval (CI): 3.42, 12.89). Subgroup analyses showed that the ORs were 4.77 (95% CI: 2.69, 8.46) in Western countries, 22.19 (95% CI: 2.46, 200.37) in Asian countries, 9.70 (95% CI: 4.43, 21.20) in the age matched group, 11.41 (95% CI: 3.32, 39.18) in the sex matched group, 9.70 (95% CI: 4.37, 21.53) in the healthy control group, and 6.82 (95% CI: 4.19, 11.10) in the sample size >30. The mean plasma Hcy level from 5 case-control studies was higher than controls, and the weighted mean difference (WMD) was 6.54 (95% CI: 2.79, 10.29). Retinal artery occlusion is associated with elevated plasma Hcy levels. Our study results suggest that hHcy is probably an independent risk factor for RAO.

## Introduction

Retinal artery occlusion (RAO) is a cause of ocular morbidity that mainly affects patients older than 60 years^1^ but also occurs in young people^2,3^, and no proven therapy exists^4,5^. RAO is the blocking of trunks or branches of the retinal circulation, which leads to the ischemic infarction of the affected retinal tissue^6,7^. Central retinal artery occlusion (CRAO) always produces severe visual loss^7–9^, and branch of the retinal artery or cilio-retinal artery occlusion provokes a remarkable visual impairment and damage to the visual field^10^. Isolated visual symptoms mean that patients may first be treated by ophthalmologists, rather than directly visiting an emergency department. When patients present with RAO, ophthalmologists should ensure that they receive appropriate acute and secondary preventive treatment^8,11^ because it is an important warning of cardiovascular and cerebrovascular events^12–15^. This means that patients with incidents of RAO are at increased risk of cardiovascular disease and ischaemic stroke^15–18^. Although there are many factors, such as embolism, haemorrhage, and intraluminal thrombosis that have been suggested to play a major role in the primary mechanisms of coronary and cerebral vascular disease and RAO in common, they do not fully explain the pathogenesis of RAO. Over the last few years, Hcy has aroused a considerable amount of interest for physicians. Elevated plasma Hcy levels appeared to be clearly associated with an increased risk of cardiovascular and cerebrovascular events^19–23^, which suggests that Hcy plays a role in the pathogenesis of vascular disease, similarly in retinal artery occlusion. Recently, the association between Hcy and the risk of RAO has been reported in some studies^24–32^, but the conclusion is contradictory and inconclusive, partially due to the relatively small sample size of individual studies, sampling effects and lack of statistical power. To overcome such limitations, we conducted a meta-analysis of the published case–control studies that directly evaluated and compared Hcy and RAO.

## Results

### Literature search

**Figure 1:**
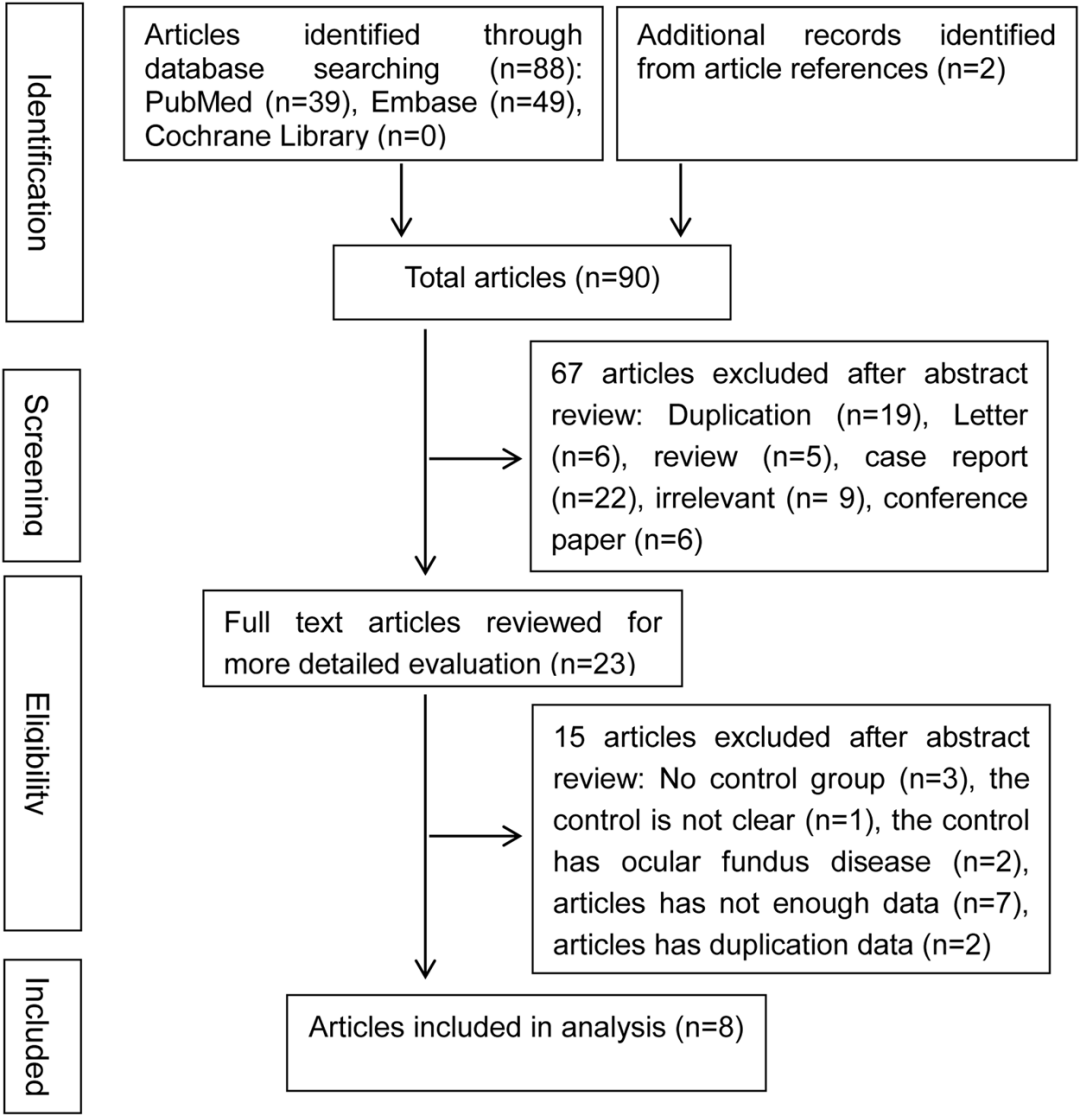
Flow chart for article selection. Flow chart shows literature search for case-control studies of RAO in relation to Hcy.

We initially searched 88 unique papers: PubMed (n = 39), Embase (n = 49) and Cochrane Library (n = 0), 2 additional publications were produced through a review of article citations. Sixty-seven studies were eliminated as follows: duplicats (n = 19), case reports (n = 22), letters (n = 6), reviews (n = 5), irrelevant (n = 9), and conference papers (n = 6). The full text of 23 potentially included papers were reviewed; we excluded three publications that did not have controls, two publications that were ocular fundus disease controls, one paper that did not have clear controls, seven papers did not have complete data, and two papers that had duplicated data. Finally, eight case-control studies were involved in our meta-analysis. The flow diagram showing the study selection is presented in Fig. 1.

### Study characteristics and quality assessment

The main information of the eight case control studies is shown in Table 1 ^24,26,28–30,32–34^. The studies were published between 2000 and 2015. These studies were performed in the United States, Europe, and Asia. The controls were healthy and hospital based individuals without RAO were included. The sample size of the studies ranged from 8 to 105, and the total number of cases was 307 in the hHcy group and 196 in the concentration of plasma Hcy group, and the numbers of controls were 542 and 350, respectively. The OR varied from 2.53 to 85.5, and the WMD ranged from 1.9 μM to 11.99 μM. All NOS scores were greater than 5, showing that the methodological quality was good.

**Table 1:** Characteristics of enrolled case-control studies of homocysteine and RAO.

Ref.	Country	Case						Control						OR	Matching	NOS score
		Source	n	Age (years)	Sex (M/F)	Hcy (μM)	hHcy	Source	n	Age (years)	Sex (M/F)	Hcy (μM)	hHcy			
34	USA	OP	74	54 ± 16	27/47	NA	13	H	110	NA	NA	NA	5	NA	NA	6
26	Ireland	CP	26	66.8	19/7	12.9	NA	HB	87	70.2	36/51	10.7	NA	2.53 (1.02−6.29)	NA	6
33	Italy	CP	41	69.6 ± 12.8	20/21	14.4 (9–53)	20	H	100	69.5 ± 11.9	58/42	11 (6.0–59)	9	9.6 (3.8–24.1)	Age, sex	9
29	Turkey	NA	20	55.8 (42–70)	12/8	21.23 ± 9.53	NA	HB	20	55.3 (50–68)	9/11	12.52 ± 4.97	NA	NA	age	6
24	Saudi	CP	8	47.1 ± 16.2	NA	20.95 ± 6.9	6	H	59	46.1 ± 11.95	44/15	8.96 ± 5.6	2	85.5 (7.49–1173.1)	Age, sex	8
32	Austria	CP	105	69.1 ± 10.6	59/46	12.2 ± 4.8	20	HB	105	68.9 ± 10.6	59/46	10.3 ± 3.4	5	4.7 (1.5–15.1)	Age, sex	8
28	UK	CP	10	69.8 ± 6.74	NA	18.4 ± 1.5	NA	H	85	51.5 ± 15.4	NA	9.5 ± 1.5	NA	NA	NA	6
30	Israel	CP	53	69.38 ± 8.65	NA	11.88 ± 3.59	26	H	81	66 ± 18	NA	8.7 ± 4.5	8	NA	Age	7

Hcy: plasma homocysteine level; hHcy: number of hyperhomocysteinemia; n: number; NA: not available; M: male; F: female; HB: hospital-baed controls; H: healthy; CP: consecutive patient; OP: outpatient; Case: patients with retinal artery occlusion; Control: without retinal artery occlusion.

### hHcy and the risk of RAO

**Figure 2:**
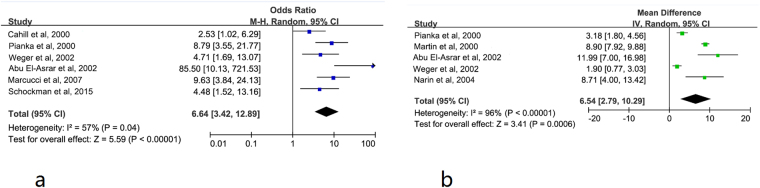
Forest plot of ORs for hHcy and RAO (**a**), and WMDs for hcy and RAO (**b**). The blue square is the OR value, the green square is the WMD value,the black diamond is the 95% CI value. The last black square is the pooled OR or WMD.

Figure 2a ^24,26,30,32–34^ shows the relationship of hHcy and the risk of RAO using the random effects model of combining OR. Six studies, including 307 RAO patients and 542 controls, have assessed the proportion of hHcy^24,26,30,32–34^. The rate of hHcy was significantly greater than the controls (OR: 6.64 [95% CI: 3.42, 12.89]; *P* < 0.00001). Substantial heterogeneity was observed (*P* = 0.04, I^2^ = 57%).

### Plasma levels of Hcy and the risk of RAO

Figure 2b ^24,28–30,32^ shows the results of the random-effects models of combining WMD for the average plasma Hcy level of the RAO patients and controls in 5 studies^24,28–30,32^, and a total of 196 cases and 350 controls were included in the meta analysis. The results showed that the plasma Hcy level is higher than the controls (WMD: 6.54 μM [95% CI: 2.79, 10.29]; *P* = 0.0006). It indicates that the increased plasma Hcy might be an independent risk factor for RAO. There was evidence of heterogeneity for this outcome (*P* < 0.00001, I^2^ = 96%).

### Sensitivity analyses

**Figure 3:**
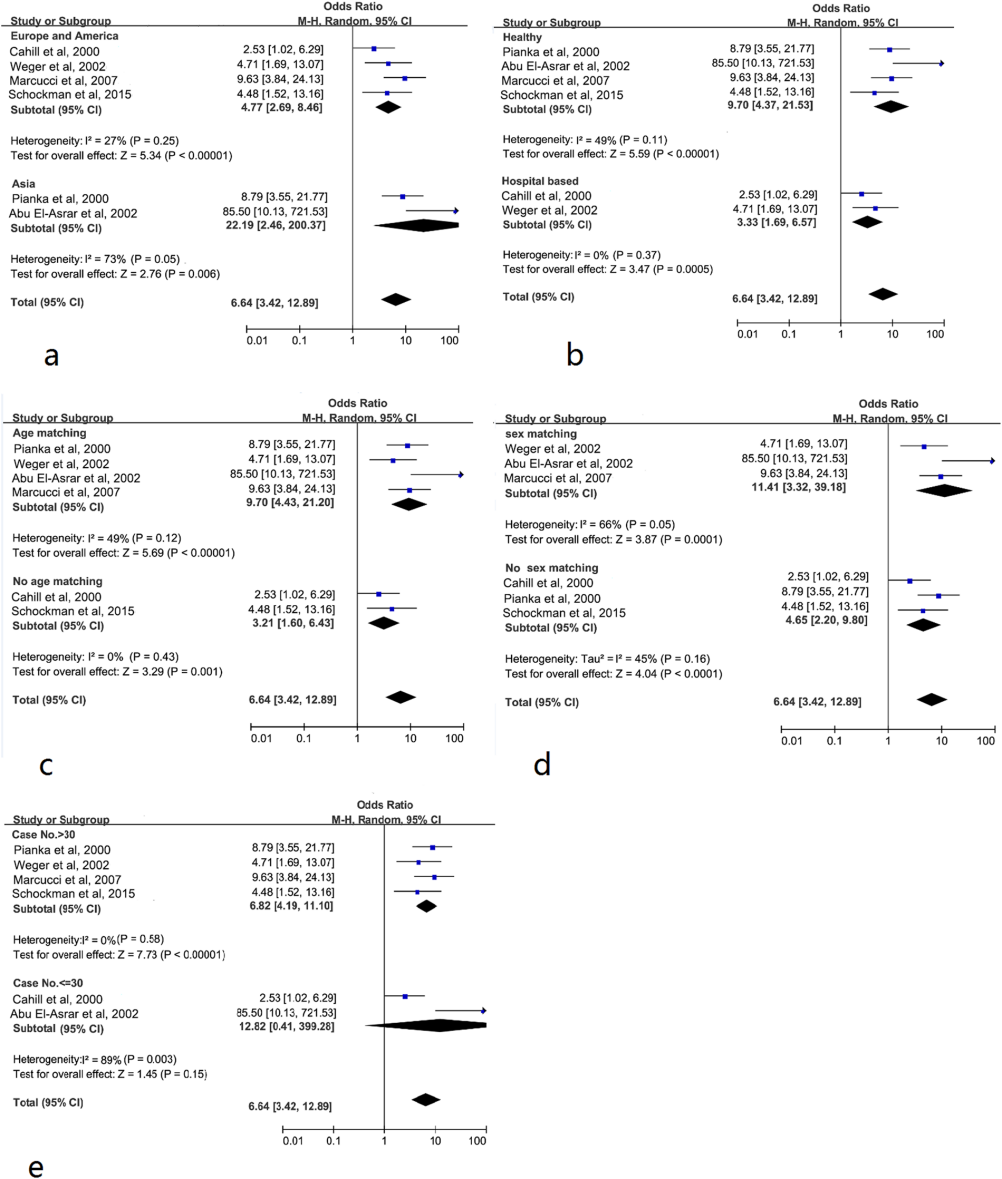
Forest plot of the subgroup analysis of ORs for hHcy and RAO. The geographical region (**a**), the characteristic of control healthy state (**b**), age matching (**c**), sex matching (**d**), and the sample size (**e**) The blue square is the OR value, the black diamond is the 95% CI.

There was significant heterogeneity among the six studies concerning the relation between hHcy and RAO; thus, we conducted subgroup analysis and sensitivity analysis. The region, sex, age, the selection of controls, and the number of included RAO patients may influence the summary combination, so we performed subgroup analysis for these factors. According to region, the studies were divided into two subgroups, and significant heterogeneity was found in the Asian region (*P* = 0.05, I^2^ = 73%), while little heterogeneity was found in the European and American region (*P* = 0.25, I^2^ = 27%). The pooled ORs were 4.77 [95% CI: 2.69, 8.46] in the European and American Region and 22.19 [95% CI: 2.46, 200.37] in the Asian Region respectively (Fig. 3a)^24,26,30,32–34^. The high OR indicates that the patients with RAO had higher incidences of hyperhomocysteinemia than the control group. In this study, the results illustrate that the RAO population has a higher rate of hyperhomocysteinemia in Asian countries compared with European and American countries. Because the controls may have affected the result, another subgroup analysis was performed, which was divided into the healthy controls and hospital-based controls (Fig. 3b)^24,26,30,32–34^, and no substantial heterogeneity was found in both subgroups respectively. According to whether age-matching or sex-matching was performed in the control group, the trails were separated into two subgroups (Fig. 3c and d)^24,26,30,32–34^. The heterogeneity was still significant in sex-matched subgroup (*P* = 0.05, I^2^ = 66%), while insignificant in the age-matching subgroup (*P* = 0.12, I^2^ = 49%). According to the RAO inclusion number, it was divided into two subgroups. Two trails^24,26^ that enrolled patients less than 30 were excluded, but this did not change the overall risk estimate (OR: 6.82 [95% CI: 4.19 to 11.10]); no evidence of heterogeneity was observed among the remaining studies (*P* = 0.58, I^2^ = 0%), as shown in Fig. 3e ^24,26,30,32–34^. Heterogeneity was still present through subgroup analysis; the overall combined OR was not substantially altered. Furthermore, the exclusion of a single study and its effect on the overall OR estimate was evaluated. The combined OR ranged from 5.45 (95% CI: 3.29, 9.01; *P* < 0.00001) to 8.15 (95% CI: 4.23, 15.71; *P* < 0.00001), and each study did not significantly affect the summary estimate, which suggested the high stability of the result. The heterogeneity was reduced when study^24^ or^26^ was eliminated. The above analyses consistently illustrate that there was a significantly positive association between hHcy and RAO.

Substantial heterogeneity was observed among the 5 studies, which showed the association between plasma Hcy and RAO. Thus, a sensitivity analysis was performed to explore the potential source. It exhibited lower results with lower heterogeneity (WMD: 2.41 μM [95% CI: 1.54, 3.82], *P* < 0.00001, I^2^ = 50%) while excluding three studies^24,28,29^ that included RAO patients less than 30. We also performed sensitivity analysis by omitting one study each time, and the combined WMD ranged from 5.28 μM [95% CI: 2.36, 8.2, *P* = 0.0004] to 7.84 μM [95% CI: 3.8, 11.88, *P* = 0.004]. This yielded a similar result with similar heterogeneity.

### Publication bias

**Figure 4:**
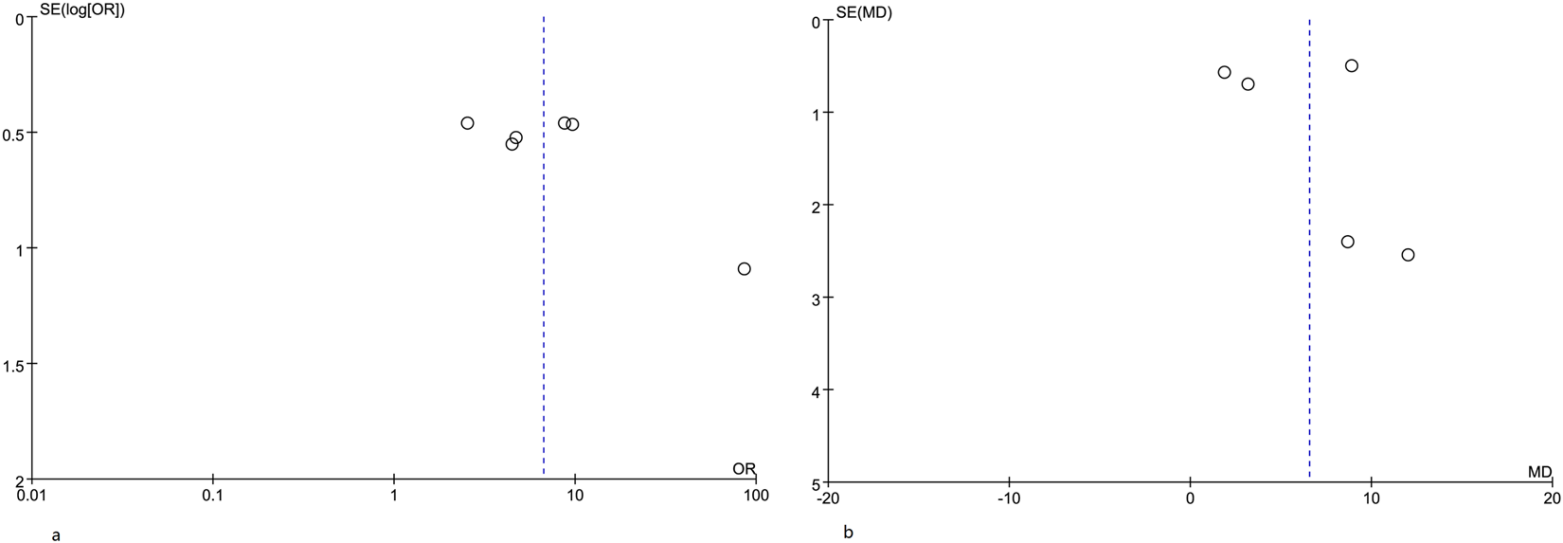
Funnel Plot detailing publication bias in the studies reporting the impact of hHcy on RAO (**a**), and mean levels of Hcy on RAO (**b**). The X axis is the ORs value, the Y axis is the log ORs value in a. The X axis is the WMDs value of Hcy, the Y axis is the WMDs standard error value in b. The cycles indicate the value of each study.

Visual inspection of the Begg funnel plot did not identify substantial asymmetry (Fig. 4a,b). The fail-safe number (N_fs_) was calculated. The N_fs_ values were 24.44 in the hHcy group and 136.10 in the plasma Hcy group. The N_fs_ was bigger than the number of observed studies included in the meta-analyses, which implied a non-significant publication bias.

## Discussion

Thrombophilia is a complex hypercoagulable state that increases the risk of thrombosis. It plays a significant pathoetiologic role in the development of RAO, which is associated with stroke, coronary artery disease, atrial fibrillation, and carotid stenosis^35^. The risk factors of thrombophilia include hyperlipidemia, arteriosclerosis, homocysteine and so on^36,37^. However, the association between homocysteine and the risk of RAO has not been fully understood, and consistent conclusions have not been provided. Some articles have suggested that higher plasma Hcy levels were associated with the risk of RAO^26^, while some reports indicated that there is no significant difference among them^25,27^. Therefore, we performed a meta-analysis to provide a quantitative assessment of the relationship. Our meta-analysis of eight case-control studies demonstrates that hHcy or higher plasma Hcy concentrations will increase the risk of RAO. Moreover, in an attempt to produce more stable, robust and credible results, we performed a subgroup analysis and a sensitivity analysis, and the results did not materially change the pooled results. Therefore, our results displayed that the elevated plasma level of Hcy or hHcy is an independent risk factor for RAO. The estimation of plasma Hcy levels should be considered in patients with RAO. The identification of elevated plasma Hcy levels may also reduce the patient’s risk of developing a cardiovascular event because retinal emboli are an important predictor of stroke and other vascular diseases^27,38^.

The underlying mechanisms may explain why Hcy can increase the risk of RAO. On the one hand, Hcy-induced endothelial cell dysfunction, endothelial cell apoptosis and proliferation disorders were exacerbated^39^, Hcy significantly increased inducible nitric oxide synthase and endothelial nitric oxide synthase and decreased glutathione levels indicating oxidative-nitrosative stress. On the other hand, the increased expression of matrix metalloproteinase (MMP) and the decreased expression of tissue inhibitor of metalloproteinase (TIMP) and tight junction proteins (zonula occluden-1) indicate vascular remodelling. Hcy promots the expression of profibrogenic cytokines and influences the MMP/TIMP balance^40^. The MMP is known to regulate the activities of collagenase and gelatinase^41^. MMP degrades collagen, allows for smooth muscle cell migration within a vessel, and induces vascular remodeling^42^.

### Sources of heterogeneity

It was not surprising that there was substantial heterogeneity among the publications in this meta-analysis (I^2^ > 50%). Several factors might explain the heterogeneity, such as the characteristics of populations, the source of populations, and the adjustment of confounding factors. Our analysis suggests that two studies^24,26^ with small sample sizes probably contribute to the heterogeneity and the heterogeneity will dispear (I^2^ = 0) after delete these two articles (Fig. 3e). As we all know that the incidence of RAO is associated with age^43^, sex, systemic disease^18,44^ and so on, so we did subgroup analysis in these side. And we found age-matching (Fig. 3c), geographic region (Fig. 3a) and healthy controls (Fig. 3b) can decrease the heterogeneity. No substantial heterogeneity was founded in the western country (Fig. 3a, I^2^ = 27%).

### Study strengths and limitations

The major strength of this study is the comprehensiveness of literature retrieval and review. Its data are as complete as possible, and we included only original case-control studies. Moreover, we performed a subgroup analysis and sensitivity analysis to further illustrate the result of this topic. In addition, with accumulating evidence and enlarged sample sizes, we have enhanced the statistical power to provide more precise and reliable risk estimates. The results of the synthesis data would provide a reference for clinical doctors. However, our study also has several limitations. Firstly, all studies were retrospective study and did not include blinding and randomization, which may have resulted in bias. Secondly, the geographic region was limited in American^34^, European^26,28,29,32,33^, and Asian^24,30^ countries, so the result were restricted universal to other regions. Thirdly, because RAO is a multi-factorial disorder, it is associated with age, sex, and systemic disease. Not every study conducted the confounding factors. Fourthly, there is substantial heterogeneity among studies. We performed subgroup analysis and sensitivity analysis, but heterogeneity still exists. Fifthly, the sample size was small. Even considering limitations, the results of these outcomes were consistent. Finally, although little evidence of publication bias was observed, the statistical power of these tests was limited due to a relatively small number of included clinical trials. However, the aforementioned limitations may affect the result, so further well-designed and well-conducted studies are needed to validate the relationship between Hcy and RAO.

In conclusion, the basis of our assessment suggests that Hcy significantly improves the risk of RAO and that it is probably an independent risk factor. The physician should estimate plasma Hcy levels when patients exhibit RAO, which may reduce the patient’s risk of developing a cardiovascular event.

## Materials and Methods

We attempted to follow the proposed MOOSE (Meta-Analysis of Observational Studies in Epidemiology) group guidelines^45^ to report the present meta-analysis.

### Literature search strategy

We searched the following databases: PubMed, EMBASE through the OVID platform, and CoChrance Library. No languages or publication year were restricted; the final search was carried out in April, 2016. Literature retrieval was conducted to identify all relevant trials that compared the association between Hcy and RAO. The following terms were used as key words or Mesh words: “homocysteine” OR “hyperhomocysteinemia” and “retinal artery occlusion” OR “retinal artery thrombosis”. In addition, we searched manually on the reference lists of retrieved articles and recent reviews for additional relevant articles to identify possible relevant articles.

### Inclusion criteria

Studies that were considered eligible in this meta-analysis met the following criteria: (1) The study design was a case-control study. (2) The interesting exposure was plasma Hcy level or hHcy, and the interesting outcome was RAO. (3) Plasma Hcy concentrations or ORs and their 95% CIs or data were used to calculate them. (4) If multiple publications for the same study were available, we used the most recent study. If necessary, we used data from the early publications. (5) The control was healthy or hospital-based patients with other eye disease. (6) The control had no ocular fundus disease.

### Exclusion criteria

**(**1) Editorials, letters to the editor, case reports, previous meta-analyses, review articles, and meeting abstracts. (2) The control group was not clearly defined. (3) Incomplete raw data.

### Data Extraction and Quality Assessment

Two authors (Xuetao Huang and Yezhen Yang) independently conducted the study selection and data extraction; any disagreements were resolved by discussion, and the authors reached a consensus on all details. The following information was extracted from each article: first authors, year of publication, country, age and sex, the number of cases and controls, the Hcy levels in the cases and control subjects, the number of hHcy in the cases and controls, the OR of the association between hHcy and RAO. We assessed the quality of each study using the Newcastle-Ottawa Scale (NOS)^46^. The NOS uses a “star” rating system to judge methodological quality. The scores range from 0 to 9, with scores 0–4 indicating low quality and scores 5–9 indicating high quality.

### Sensitivity analysis

Because the characteristics of populations, the diagnosis of RAO, the source of controls and adjusting confound factors varied among all studies, we further conducted a subgroup analysis and sensitivity analysis to explore the possible source of heterogeneity. We also assessed the stability of the results by removing a single study each time to recalculate the pooled data.

### Statistical analysis

We used Review Manager 5.2.6 to calculate the pooled data. The odds ratios (ORs) were used as a common measure to evaluate the association between hHcy and the risk of RAO. The weighted mean differences (WMDs) were used to compare the plasma Hcy concentrations between the case and control subjects. The pooled data were calculated by using a random-effects model to achieve a more conservative assessment.

Statistical heterogeneity was estimated using Cochran’s Q test and the I^2^ statistics. For the Q statistic, *P* < 0.1 was considered statistically significant heterogeneity among the combined studies. I^2^ values of 50% or more indicate a significant heterogeneity.

Publication bias was qualitatively assessed by the visual inspection of funnel plot asymmetry of the logarithmic OR against their standard errors and the MD against their standard errors. We also used N_fs_ to assess the presence of publication bias, if the N_fs_ is bigger, the results are more stable, and the results are more credible. If the value of N_fs_ for one comparison turned out to be smaller than the number of included studies, it showed a significant publication bias. The N_fs_ is calculated by the formula N_fs_ = [ΣZ(p*i*)]^2^/1.645^2^ − n, where n equals the number of observed trials, and p = 0.05.
